# Artificial Intelligence-Powered Interpretation of Corneal Epithelial Maps: A Comparative Pilot Study of ChatGPT, Google Gemini, and Microsoft Bing

**DOI:** 10.7759/cureus.90779

**Published:** 2025-08-22

**Authors:** Ruchi Shukla, Aparajita Shukla, Ashutosh K Mishra, Pragati Garg, Nilakshi Banerjee, Swarastra P Singh, Shrinkhal

**Affiliations:** 1 Ophthalmology, All India Institute of Medical Sciences, Raebareli, Raebareli, IND; 2 Neurology, All India Institute of Medical Sciences, Raebareli, Raebareli, IND

**Keywords:** artificial intelligence, corneal epithelial mapping, keratoconus, pterygium, vernal keratoconjunctivitis

## Abstract

Background

This study aimed to compare the diagnostic interpretation accuracy and clinical suitability of three generative artificial intelligence (AI) models, i.e., ChatGPT 4.0, Google Gemini, and Microsoft Bing, in analyzing corneal epithelial thickness (CET) data across key ocular surface disorders, including keratoconus, vernal keratoconjunctivitis (VKC), and nasal pterygium.

Methodology

Standardized case scenarios with corresponding CET mapping data were constructed and input into all three AI platforms with the following query: “Evaluate the given CET map and provide the most likely diagnosis and appropriate clinical recommendation.” Responses were independently graded by a panel of three ophthalmologists for diagnostic accuracy and clinical appropriateness. Cases were selected based on known CET signature patterns derived from the literature, including doughnut patterns in keratoconus, superior thinning in VKC, and nasal epithelial thickening in nasal pterygium.

Results

Of the 15 AI-evaluated case scenarios (five each of keratoconus, VKC, and nasal pterygium), ChatGPT showed the highest diagnostic accuracy (80%) and clinical appropriateness (87%). Google Gemini correctly diagnosed 60% and was deemed clinically appropriate in 67%. Microsoft Bing yielded 53% correct diagnoses and 60% appropriate clinical suggestions.

Conclusions

ChatGPT 4.0 consistently outperformed Google Gemini and Microsoft Bing in the context of CET interpretation for common ocular surface diseases. These findings suggest that ChatGPT may serve as a valuable adjunct in AI-assisted ophthalmology diagnostics, particularly for ocular surface diseases where subtle epithelial remodeling is crucial for early identification. While diagnostic accuracy was the primary outcome, appropriateness of suggested appropriate clinical recommendations aligned with standard protocols in the majority of ChatGPT responses (87%), highlighting its clinical utility.

## Introduction

The thin outermost layer of the cornea, known as the corneal epithelium, is thought to be crucial for preserving the integrity of the cornea [1]. Because of its capacity for regeneration, the epithelium is continuously going through a process of renewal through the proliferation of limbal stem cells [2]. The entire corneal epithelium takes four to seven days to fully recover.

The corneal epithelium’s thickness is surprisingly uniform in a normal population, and under the impact of limbal stem cell renewal and regeneration, the thickness profile stays constant [3]. However, in corneal diseases, the corneal epithelium’s thickness varies. The underlying stromal abnormalities and asymmetries are thought to be extremely responsive to the corneal epithelium [4].

Corneal epithelial thickness (CET) analysis plays a crucial role in the early identification and assessment of various ocular surface diseases [5]. CET mapping has demonstrated diagnostic relevance in conditions such as epithelial basement membrane dystrophy (EBMD), limbal stem cell deficiency (LSCD), keratoconus, and dry eye disease [6].

In EBMD, CET mapping may reveal focal irregularities due to basement membrane abnormalities, with entrapped epithelial cells and thickened membrane patterns causing surface irregularity, blurred vision, and risk of recurrent erosions [7]. In LSCD, anterior segment optical coherence tomography (AS-OCT) reveals significant thinning of the corneal and limbal epithelium, with epithelial thickness reduction correlating with disease severity [8], making CET mapping a valuable noninvasive diagnostic tool. In dry eye disease, CET mapping reveals regional thinning and inferior-superior asymmetry, providing objective markers for disease severity and progression [9].

Keratoconus is a progressive corneal ectatic disorder characterized by uneven thinning and cone-like corneal protrubence, often starting around puberty and leading to significant vision loss requiring corneal transplantation [10]. It is a multifaceted corneal disorder influenced by genetic, environmental, and mechanical factors, with its exact etiology remaining unclear due to rapid tissue degeneration and lack of suitable animal models, but it is characterized by intricate degenerative-regenerative mechanisms and decreased corneal collagen [11]. CET mapping aids in distinguishing keratoconus from conditions such as contact lens warpage and asymmetric astigmatism, with early keratoconus showing apical thinning and a characteristic “donut pattern,” often preceding topographic changes [12].

CET mapping can reveal epithelial thinning over and around the thinnest pachymetry areas before the classic donut pattern in early keratoconus, while long-term contact lens wear shows diffuse thinning; monitoring inferior paracentral CET changes is useful for tracking disease progression [13].

Recurrent, bilateral allergic inflammation of the conjunctiva is known as vernal keratoconjunctivitis (VKC). It is a form of allergic conjunctivitis, often shows seasonal exacerbations, commonly in spring and summer, and may become perennial with disease progression. AS-OCT-based epithelial thickness mapping can detect and monitor significant superior and minimal area corneal epithelial thinning seen in children with VKC [14].

Pterygium is a chronic condition affecting the ocular surface, marked by a fibrovascular overgrowth of the conjunctiva onto the cornea, typically in a wing-like shape. Even when the visual axis is not directly involved, pterygium can still compromise visual quality by causing corneal disturbance, astigmatism, and increased higher-order aberrations [15]. Some studies have shown that pterygium is associated with a mild reduction in central corneal thickness, along with significant alterations in corneal curvature, tear volume, and visual acuity [16].

The integration of artificial intelligence (AI) in corneal epithelial mapping is revolutionizing the diagnostic landscape for ocular surface disorders. AI algorithms, when applied to epithelial thickness maps generated by AS-OCT, can detect subtle remodeling patterns and asymmetries with higher precision. This is particularly beneficial in conditions such as VKC, pterygium, and keratoconus, where epithelial alterations precede overt stromal changes. As spectral-domain OCT (SD-OCT) offers a rapid, non-contact, and non-invasive method for generating high-resolution epithelial thickness maps [17], it forms an ideal platform for AI-based analysis. By enhancing the sensitivity and specificity of early disease detection and progression monitoring, AI-powered assessment holds promise for personalized management and surgical decision-making in corneal practice.

Although several studies have investigated CET using SD-OCT, there remains a significant gap in the literature regarding the application of AI to these datasets. To date, no studies have comprehensively compared or evaluated corneal epithelial mapping results using AI algorithms in conditions such as VKC, pterygium, and keratoconus. Given that SD-OCT offers a non-invasive, high-resolution method for imaging ocular tissues, integrating AI could enhance the sensitivity and objectivity of detecting subtle epithelial changes, especially in early or subclinical stages of disease.

With the surge in healthcare-related AI applications, generative AI models such as ChatGPT, Microsoft Bing, and Google Gemini offer potential for diagnostic support. Microsoft Bing, while primarily a search engine and browser, integrates a conversational AI interface (Copilot) powered by GPT-4. For this study, Microsoft Bing was considered in its chat interface capacity, not as a standalone AI model. While prior studies have tested these models in neuro-ophthalmology, their ability to interpret CET-based topographic data has not been systematically compared.

## Materials and methods

In total, 15 cases of CET mapping were selected to represent distinct clinical conditions, including keratoconus, VKC, and nasal pterygium. Typical keratoconus cases were characterized by inferotemporal epithelial thinning and classic doughnut-shaped patterns as the epithelium thins in the inferotemporal area, with compensatory thickening occurring around it. VKC cases exhibited superior CET thinning, primarily attributed to mechanical rubbing of the eyelid over the superior part of the corneal epithelium, while nasal pterygium cases demonstrate nasal CET thickening due to localized inflammation and fibrosis. For each case, comprehensive data were collected, including demographic information, presenting symptoms, and SD-OCT-based CET map descriptors, to support detailed analysis and interpretation.

All three AI models were prompted with the following standardized query: “Given the following CET map and clinical symptoms, what is the most likely diagnosis?” To ensure impartiality and prevent any carryover effects or contextual bias, each prompt was entered in a separate, newly initiated chat instance. It was entered via a unique IP-masked browser session to minimize potential model bias from prior conversation history.

Inclusion criteria were patients aged 18 years and above, CET maps from eyes clinically diagnosed as keratoconus, VKC, or nasal pterygium based on slit-lamp and SD-OCT findings, availability of high-resolution epithelial maps with associated clinical symptoms, and only high-quality CET maps with clear visibility, strong signal strength, and complete corneal coverage were included to ensure reliable interpretation. This ensured consistency and minimized diagnostic errors due to poor imaging.

The following exclusion criteria were applied: poor-quality OCT scans or incomplete CET maps; ambiguous or mixed pathology where diagnosis could not be clearly attributed to a single disorder; cases with previous corneal surgery, trauma, or concurrent infections; and cases with nasal pterygium in locations other than the nasal to ensure epithelial thickness mapping uniformity.

**Figure 1:**
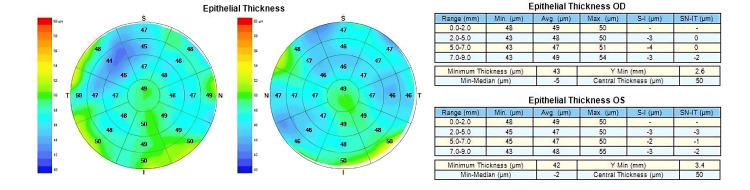
Corneal epithelial thickness pattern in the left eye with Grade 2 nasal pterygium.

**Figure 2:**
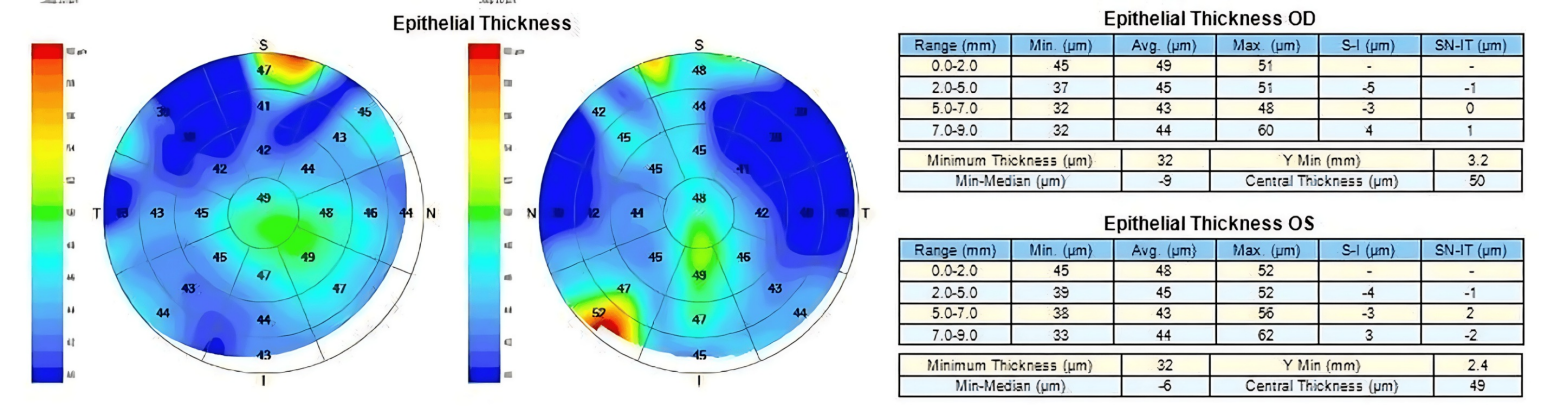
Corneal epithelial thickness patterns in both eyes of a patient with active vernal keratoconjunctivitis.

**Figure 3:**
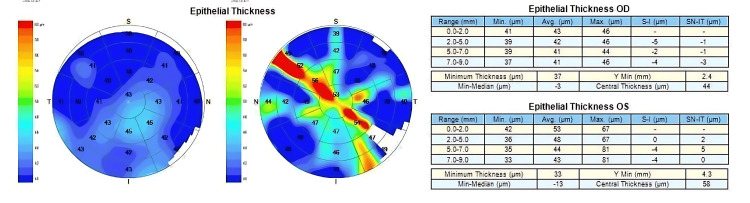
Corneal epithelial thickness pattern in the left eye with unilateral keratoconus.

Figures 1-3 are examples of case images that were input into the three large languages. Figure 1 is the input image of the CET pattern in the left eye with Grade 2 nasal pterygium. Figure 2 is the input image of the CET patterns in both eyes of a patient with active VKC. Figure 3 is the input image of the CET Pattern in the left eye with unilateral keratoconus.

The following two evaluation metrics were used: diagnostic accuracy - whether the AI-generated diagnosis matched the actual known clinical diagnosis; and clinical appropriateness - whether the AI response reflected standard ophthalmological management, including language consistency and logical justification. All responses were independently graded by a cornea specialist, who was blinded to the identity of the AI platform.

On statistical analysis, the diagnostic accuracy and clinical appropriateness were calculated as percentages for each model across all three disorders. Descriptive statistics (frequency and percentage) were used to present the performance outcomes of each model. Comparative performance was visualized using bar graphs to highlight inter-model variation by condition. The level of agreement for accuracy and appropriateness among three AI tools was calculated with the help of Cohen’s kappa statistic.

## Results

A comparative assessment of three AI models, ChatGPT, Google Gemini, and Microsoft Bing, was conducted using cases with similar severity that were selected to evaluate the diagnostic accuracy and clinical appropriateness of the AI models in interpreting CET patterns across three anterior segment disorders, i.e., keratoconus, VKC, and nasal pterygium. Table 1 shows a few examples of actual clinical diagnoses and their comparison with the results of the AI models.

**Table 1:** Diagnoses generated by the three AI models based on the input images and corresponding clinical scenarios. AI: artificial intelligence; CET: corneal epithelial thickness; SN-IT: superonasal-inferotemporal

Case number	Actual diagnosis	ChatGPT	Gemini	Microsoft Bing
1	CET pattern in the left eye with Grade 2 nasal pterygium	CET map shows mild nasal epithelial thickening with overall symmetrical distribution and minimal SN-IT difference, indicating no ectatic pattern. These findings are consistent with localized mechanical or inflammatory changes seen in pterygium	CET map shows nasal epithelial thickening with symmetrical distribution and no significant SN-IT asymmetry, suggesting epithelial remodeling due to pterygium. Differential diagnoses include chronic allergic conjunctivitis, early OSSN, or pinguecula	Early keratoconus or dry eye disease, based on subtle inferotemporal epithelial thinning and symptoms. Differential diagnoses include contact lens-related epitheliopathy, post-LASIK remodeling, or epithelial basement membrane dystrophy
2	CET patterns in both eyes of a patient with active vernal keratoconjunctivitis	Bilateral corneal epithelial remodeling with superior thinning, consistent with vernal keratoconjunctivitis	Irregular and patchy corneal epithelial remodeling observed on the CET map strongly points toward allergic conjunctivitis as the most likely diagnosis. The epithelial changes are likely a secondary manifestation of the chronic inflammation, rubbing, and tear film instability caused by the allergic reaction	Allergic conjunctivitis or dry eye syndrome, based on symptoms and CET findings, but pinguecula/pterygium or infectious conjunctivitis should be ruled out with slit-lamp evaluation
3	CET pattern in the left eye with unilateral keratoconus	Unilateral keratoconus (left eye) with classic epithelial doughnut pattern	This kind of highly irregular and often thin epithelial pattern (especially the left eye with its distinct thick and thin areas) is indicative of significant epithelial remodeling. Common reasons for such epithelial remodeling include corneal ectatic diseases, prior refractive surgery, and chronic dry eye disease	Most likely diagnosis include leratoconus, based on inferior corneal thinning seen in the CET map

Each model was assessed using five standardized clinical vignettes (n = 5 per disorder), with performance metrics expressed as percentages. Table 2 shows the comparison of accuracy and appropriateness in percentage on comparing the three AI models for all 15 cases. On analyzing the agreement for accuracy and appropriateness between ChatGPT, Google Gemini, and Microsoft Bing, results were statistically significant between Google Gemini and Microsoft Bing in pterygium cases (Table 3).

**Table 2:** Metrics for all three disorders in terms of both suitability and accuracy, as provided by the three AI models. AI: artificial intelligence; KCN: keratoconus; VKC: vernal keratoconjunctivitis

Disorder	Metric	ChatGPT (%)	Google Gemini (%)	Microsoft Bing (%)
KCN	Accuracy	100	80	60
	Appropriateness	100	80	60
VKC	Accuracy	60	40	40
	Appropriateness	80	60	60
Pterygium	Accuracy	80	60	60
	Appropriateness	80	60	60

**Table 3:** Agreement for accuracy and appropriateness between ChatGPT, Google Gemini, and Microsoft Bing. Values in respective cells represent Cohen's kappa values and those in split cells represent p-values. ^#^: No statistics are computed, ChatGPT accuracy is a constant. ^!^:  Statistics are not computed in cells which compare one AI model with itself. ^*^: p-values <0.05 are considered statistically significant. AI: artificial intelligence; CET: corneal epithelial thickness; KCN: keratoconus; VKC: vernal keratoconjunctivitis

	Accuracy			Appropriateness		
	ChatGPT	Google Gemini	Microsoft Bing	ChatGPT	Google Gemini	Microsoft Bing
KCN						
ChatGPT	NA^!^	NA^#^	NA^#^	NA^!^	NA^#^	NA^#^
Google Gemini	NA^#^	NA^!^	0.545	NA^#^	NA^!^	0.545
			0.17			0.17
Microsoft Bing	NA^#^	0.545	NA^!^	NA^#^	0.545	NA^!^
		0.17			0.17	
VKC						
ChatGPT	NA^!^	0.615	-0.15	NA^!^	0.545	0.545
		0.136	0.709		0.171	0.171
Google Gemini	0.615	NA^!^	0.167	0.545	NA^!^	0.167
	0.136		0.709	0.171		0.709
Microsoft Bing	-0.15	0.167	^!^NA	0.545	0.167	NA^!^
	0.709	0.709		0.171	0.709	
Pterygium						
ChatGPT	NA^!^	0.545	0.545	NA^!^	0.545	0.545
		0.171	0.171		0.171	0.171
Google Gemini	0.545	NA^!^	1.00	0.545	NA^!^	1.00
	0.171		0.025^*^	0.171		0.025^*^
Microsoft Bing	0.545	1.00	NA^!^	0.545	1.00	NA^!^
	0.171	0.025^*^		0.171	0.025^*^	

**Figure 4:**
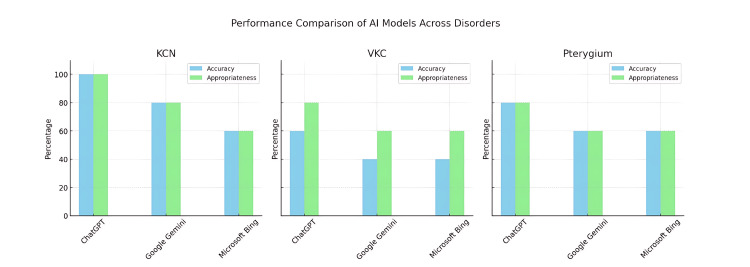
Comparative performance of AI models (ChatGPT, Google Gemini, Microsoft Bing) in diagnosing ocular surface disorders based on CET patterns. AI: artificial intelligence; CET: corneal epithelial thickness; KCN: keratoconus; VKC: vernal keratoconjunctivitis

Figure 4 illustrates the comparative performance across all three AI models, separated by diagnostic category and performance metric (accuracy and appropriateness). ChatGPT consistently outperformed its counterparts, especially in keratoconus cases where epithelial remodeling follows a distinct central thinning pattern. The chart highlights the diminishing diagnostic accuracy across all models in VKC, reflecting the inherent challenge of detecting nonspecific or diffuse epithelial change.

## Discussion

Our study underscores the emerging role of large language models, such as ChatGPT, in assisting with the interpretation of CET maps for the differential diagnosis of anterior segment disorders. The analysis revealed that ChatGPT outperformed other models in overall diagnostic accuracy, particularly in conditions with well-characterized epithelial profiles such as keratoconus. This superior performance can be attributed to the model’s advanced contextual reasoning and its ability to integrate complex spatial thickness data with clinical descriptors [18].

In contrast to previous literature relying mainly on convolutional neural network (CNN)-based image segmentation, our study evaluated AI models using descriptive CET features and structured prompts, offering a novel approach to anterior segment AI application [19].

An important insight from our work is the marked improvement in diagnostic output when the AI models were provided with condition-specific symptom prompts. For instance, in cases of nasal pterygium, the addition of symptoms such as nasal conjunctival growth led to a more precise interpretation of the peripheral epithelial thinning patterns typically associated with the lesion. Similarly, in VKC, prompting the model with symptoms such as itching, conjunctival congestion, and watering improved its ability to correlate clinical findings with diffuse epithelial irregularities despite the inherent variability and overlapping features with dry eye disease.

However, the performance of all three evaluated models diminished when dealing with conditions such as VKC, which exhibit highly variable and often nonspecific epithelial thickness patterns. This highlights a current limitation of general-purpose AI models: their reduced diagnostic sensitivity in scenarios where data patterns are subtle, heterogeneous, or contextually dependent. These findings reinforce the need for ophthalmology-specific fine-tuning of such models, possibly through curated clinical datasets and reinforcement learning with expert feedback.

Another notable observation was the variability in the interpretation of central versus peripheral CET changes. While most models performed reasonably well in recognizing central thinning in keratoconus or central thickening in early epithelial remodeling disorders, they were less consistent in detecting peripheral changes linked to inflammatory or degenerative etiologies-suggesting a gap in training data diversity.

In addition to identifying primary diagnoses, AI models demonstrated potential in suggesting differential diagnoses that were clinically plausible, even when not entirely accurate. This capability can broaden the diagnostic thinking process, especially for early-career clinicians.

Our study demonstrates that AI models, when supported with well-structured clinical prompts, offer promising potential in CET-based screening and preliminary diagnosis of ocular surface disorders. Future work should focus on developing domain-specific AI tools trained on large ophthalmic datasets and capable of integrating multimodal inputs, including symptoms, topography, and slit-lamp findings, to enhance clinical utility and accuracy in real-world settings.

Limitations include the absence of actual image-based CET map inputs, and reliance on descriptive data may underestimate AI models’ potential. Moreover, AI-based tools remain dependent on clinician oversight, as disease profiles, population dynamics, and technologies continue to evolve [20]. As highlighted in prior studies, concerns such as model generalizability, healthcare infrastructure variability, and potential AI bias based on population or hardware differences must also be addressed [21].

In addition, adherence to standardized AI reporting guidelines such as CLAIM, CONSORT-AI, and FUTURE-AI is essential to ensure scientific rigor, reproducibility, and transparency in AI model development and reporting in ophthalmology. These frameworks help authors present complete and clinically applicable AI research, thus facilitating review, validation, and eventual clinical adoption [22].

With the advent of multimodal models such as GPT-4o, which can process both image and text inputs, future applications may include direct image-based interpretation of CET maps, enabling even more autonomous diagnostic suggestions.

In summary, while AI models are not yet a substitute for clinical judgment, their ability to assist in pattern recognition and differential diagnosis based on CET maps represents a significant advancement in ophthalmic diagnostics. Integration of multimodal inputs, including imaging, symptomatology, and clinical history, into tailored AI frameworks may pave the way for more accurate, accessible, and standardized ocular surface disease screening in the near future.

Future research should focus on integrating actual epithelial thickness map images with advanced vision-language AI models to enhance the precision and applicability of diagnostic interpretations. Additionally, cross-validation of AI-generated analyses with real-world patient outcomes and the incorporation of these tools into real-time clinical decision-support systems will be essential for practical implementation. Expanding the scope to include applications such as monitoring epithelial remodeling following refractive surgeries and improving diagnostics for dry eye disease could further broaden the clinical utility of this approach.

## Conclusions

This comparative study highlights the emerging role of large language models in the AI-assisted interpretation of CET maps for anterior segment disorders. Among the three models evaluated, i.e., ChatGPT 4.0, Google Gemini, and Microsoft Bing, ChatGPT demonstrated the highest diagnostic accuracy and clinical recommendation appropriateness, particularly in keratoconus cases where epithelial remodelling patterns were well-defined. Notably, diagnostic accuracy improved across models when symptom-specific prompts were included, underscoring the importance of integrating clinical context with imaging data. Overall, this study supports the potential utility of AI models as adjunct tools in ophthalmic diagnostics, while also identifying the need for condition-specific fine-tuning, larger annotated ophthalmic datasets, and future integration with multimodal AI systems for broader clinical applicability.
